# The Immunomodulatory Role of Syncytiotrophoblast Microvesicles

**DOI:** 10.1371/journal.pone.0020245

**Published:** 2011-05-25

**Authors:** Jennifer Southcombe, Dionne Tannetta, Christopher Redman, Ian Sargent

**Affiliations:** Nuffield Department of Obstetrics and Gynaecology, University of Oxford, John Radcliffe Hospital, Oxford, United Kingdom; Instituto Gulbenkian de Ciência, Portugal

## Abstract

Immune adaptation is a critical component of successful pregnancy. Of primary importance is the modification of cytokine production upon immune activation. With the discovery that normal pregnancy itself is a pro-inflammatory state, it was recognised that the classical Th1/Th2 cytokine paradigm, with a shift towards ‘type 2’ cytokine production (important for antibody production), and away from ‘type 1’ immunity (associated with cell mediated immunity and graft rejection), is too simplistic. It is now generally agreed that both arms of cytokine immunity are activated, but with a bias towards ‘type 2’ immunity. Many factors are released from the placenta that can influence the maternal cytokine balance. Here we focus on syncytiotrophoblast microvesicles (STBM) which are shed from the placenta into the maternal circulation. We show that STBM can bind to monocytes and B cells and induce cytokine release (TNFα, MIP-1α, IL-1α, IL-1β, IL-6, IL-8). Other cytokines are down-modulated, such as IP-10 which is associated with ‘type 1’ immunity. Therefore STBM may aid the ‘type 2’ skewed nature of normal pregnancy. We also observed that PBMC from third trimester normal pregnant women produce more TNFα and IL-6 in response to STBM than PBMC from non-pregnant women, confirming that maternal immune cells are primed by pregnancy, possibly through their interaction with STBM.

## Introduction

A pregnant woman's immune system is carefully controlled and adapted to accommodate the developing semi-allogenic fetus. Failure to appropriately adapt is associated with pregnancy problems such as spontaneous abortion or preeclampsia. The adaptation can be seen by studying maternal cytokine responses to antigens throughout pregnancy. Cytokine responses are often described as being of type 1 or type 2; type 1 cytokines such as Interferon gamma (IFNγ) and Tumor Necrosis Factor alpha (TNFα) promote cellular mediated immune responses, and type 2 cytokines such as IL-4 and IL-6 promote humoral immunity. A bias towards type 2 immunity was proposed to prevent cell mediated rejection of the fetus [1], and such changes in cytokine immunity can be observed. Often during pregnancy classical type 1 syndromes alleviate, whereas type 2 syndromes worsen. Over recent years this concept has been shown to be too simplistic [2], [3] and the inflammatory nature of normal pregnancy has become more apparent [4]. It is now generally agreed that both arms of cytokine immunity are activated, but with a bias towards ‘type 2’ immunity [5].

It is proposed that factors from the placenta can induce these essential modifications [6]. Possible modulating factors include cytokines, growth factors and enzymes [7]. These factors can often be detected in the maternal peripheral blood and are present at varying levels throughout pregnancy, and therefore have the potential to modify maternal immunity. In addition, it is known that microvesicles (<1 µm) are shed from the syncytiotrophoblast into the maternal blood [8]. These are termed syncytiotrophoblast microvesicles (STBM) and are also thought to affect maternal immunity systemically.

Many cell types release vesicles of which there are three main types: vesicles that bud directly from the cell membrane, exosomes that are derived from multivesicular bodies within the cell and apoptotic bodies, small sealed membrane vesicles that are produced from cells undergoing cell death by apoptosis [9]. They are encapsulated by a lipid bilayer, and can contain various cytoplasmic molecules, such as cytoskeletal proteins, signalling molecules, DNA and micro RNAs. The precise nature of the placental vesicles has yet to be defined, with respect to the content and proportion of vesicles, exosomes and apoptotic bodies. We, and others, can detect STBM in the circulation of women in the first trimester of pregnancy and increasing as pregnancy progresses [10], [11].

Cellular vesicles are an integral part of various immunological systems, as they carry proteins, lipids and miRNAs from their cell of origin to other target cells. They can be immune activating, for example they can carry antigens which directly stimulate T cells, transfer antigens to dendritic cells for indirect immune cell stimulation, or act independently of antigens by exposing immune cells to stimulatory factors, such as heat shock protein-70 or NKG2D ligands [12]. In contrast, they can be inhibitory, for example they can cause T cell death, inhibit dendritic cell maturation or prevent T cell killing activity, reviewed by Thery et al (2009) [12].

STBM can interact with various target cells. *In vitro*, STBM can cause disruption to endothelium [13], induce pro-inflammatory cytokine production by immune cells [10] and inhibit T cell proliferation [14], [15]. In addition, the observation that increased levels of STBM can be detected in preeclamptic women has led to the hypothesis that STBM could contribute to the vascular endothelial disruption and exaggerated inflammation observed in this disease [6].

To study the effects of STBM on target cells sufficiently large amounts of relatively pure vesicles are needed. Ideally vesicles would be obtained from the peripheral blood of pregnant women through centrifugation, however the yield is very low and preparations would be heavily contaminated with vesicles from other cell types; STBM represent only a very small proportion of the total plasma vesicle population, the predominant form being platelet derived [11]. Therefore in this study STBM have been prepared from term placentas delivered by caesarean section using three different methods designed to mimic the STBM generated *in vivo*. These are mSTBM (derived from mechanical dissection of the placenta), eSTBM (shed from placenta explants in culture) and pSTBM (perfused from the maternal side of a placenta lobe). As normal pregnancy is associated with a systemic inflammatory response we have investigated the stimulatory capacity of the three STBM preparations. We used a cytokine array panel to identify a range of cytokines and chemokines that are produced, or inhibited, when PBMC are treated with STBM. Further studies focused on seven cytokines: TNFα, Macrophage Inflammatory Protein-1alpha (MIP-1α), IL-1α, IL-1β, IL-6, IL-8 and interferon-inducible protein 10 KDa (IP-10). Intracellular cytokine staining revealed that the monocytes were solely responsible for the production of TNFα, IL-6, IL-8 and IL-1β. We have previously shown that PBMC from pregnant women are primed to produce more TNF-α in response to LPS/IFN-γ stimulation than PBMC from non-pregnant women [10]. Here, we explore if peripheral blood mononuclear cells (PBMC) from third trimester normally pregnant women are more responsive to STBM than PBMC from non-pregnant women. In addition, we show that the STBM bind to monocytes, which also phagocytose the STBM, and to a lesser extent to B cells, but only small numbers of T and NK cells are able to bind STBM.

## Methods

### Subjects

Healthy women were recruited in the third trimester of pregnancy. The mean age of the women was 32 years (range 27 to 36), 70% were nulliparous, and mean gestation was 37+3 (range 35+3 to 39+6). Ten normal pregnant women were recruited and matched to ten non pregnant women for age (+/−4 years) and parity (0, 1–3). None of the women were in labour at the time of sampling, and all had singleton pregnancies, with no known fetal abnormalities. None of the women had any significant medical history, current or recent illnesses, or were taking medication. These studies were approved by the Oxfordshire Research Ethics Committee C and informed written consent was obtained.

### STBM preparation

Three preparations of STBM were made for this study, mechanically derived STBM (mSTBM), STBM from placenta perfusion (pSTBM) and explant derived STBM (eSTBM). All placentas were from normally pregnant, healthy women undergoing elective caesarean section, without labor, and were processed immediately. Protein content was determined using a Pierce BCA protein assay kit (Thermo Scientific, Illinois, USA), and aliquots of microvesicles were stored at −80°C. STBM were cultured in antibiotic free media for five days to confirm that the preparations were not contaminated with bacteria.

mSTBM were prepared by a modification of the method of Smith et al. [16], as outlined previously [13]. Briefly, placenta tissue is washed in ice cold 100 mM CaCl_2_ followed by PBS and scraped from villi and then stirred in 0.9% NaCl buffer for 1 hour. Cell debris was removed by centrifugation in a Beckman J6-M centrifuge at 600×g for 10 min at 4°C and 10,000×g for 10 min , then the supernatant was centrifuged at 48,000×g for 45 min at 4°C in a Beckman L8-80M ultracentrifuge. The resultant pellets were pooled and washed in PBS before finally being resuspended in PBS. Typical mSTBM preparations yield 25–100 mg of vesicles. A pool of nine mSTBM preparations was made for use in all experiments.

pSTBM were prepared using a modified dual placental perfusion system as described by Eaton and Oakey [17]. An individual lobule was isolated and firstly the fetal circulation perfused with 0.1 µM filtered modified M-199 tissue culture medium (Medium 199 with L-glutamine and Earle's salts, containing 0.8% Dextran 20, 0.5% BSA, 5000 U/L sodium heparin, and 2.75 g/L sodium bicarbonate, pH 7.4) containing a 20 ml bolus of 100,000 IU streptokinase to promote clot removal, at a rate of 5 mL/min. The whole placenta was turned upside down and laid inside a Perspex water jacket maintained at 37°C. The maternal circulation was then perfused with medium (Medium 199 with L-glutamine and Earle's salts, containing 0.5% BSA, 5000 U/L sodium heparin, and 2.75 g/L sodium bicarbonate, pH 7.4) through eight 1.7 mm fetal feeding tubes at a controlled rate of 20 ml/min. Perfusion media were warmed in a 37°C water bath and the maternal perfusion medium was oxygenated with 95% O2, 5%CO2. The lobule was perfused for 20 min to equilibrate the system, after which time the maternal circuit was closed with a total volume of perfusion medium of 600 mL. The volume of fetal effluent was measured every 2 min and the oxygen concentration of the maternal side perfusate monitored to ensure the stability of the preparation. Pressure monitors were used to ensure no significant deviations from baseline during the experimental period. At the end of the 3 hr perfusion period, the maternal perfusate was centrifuged in a Beckman J6-M centrifuge at 600×g for 10 min at 4°C. The supernatant was centrifuged at 150,000×g for 1 hour at 4°C in a Beckman L8-80M ultracentrifuge. The resultant pellets were pooled and washed in PBS before finally being resuspended in PBS to give a final protein content of 5 mg/ml. Typical pSTBM preparations yield 25–50 mg of vesicles. Five pSTBM preparations were pooled for use in all experiments.

eSTBM were prepared as follows. Freshly delivered placentae were first rinsed in ice cold Hanks balanced salt solution and placed into a glove box maintained at 8% oxygen. Placental pieces, cut from undamaged lobules that appeared healthy, were rinsed in 8% O_2_ equilibrated ice cold explant culture medium (DMEM/F12 containing 10% foetal bovine serum (PAA Laboratories GmbH, Austria), 1% antibiotic and antimycotic solution (Sigma Aldrich, UK) and L-glutamine) before being placed into ice cold fresh equilibrated explant culture medium. Placental pieces of approximately 2 mm in diameter were then dissected and distributed equally between Costar Netwell (24 mm diameter, 500 µm mesh) supports in 6-well plates containing 4 ml/well equilibrated explant culture medium (10 explants/well). Placental explants were finally washed again with a medium change before being incubated under ‘normoxic’ conditions (8% O_2_/87% N_2_/5% CO_2_) [18]. After 24 hr the explant supernatant was collected and centrifuged at 600×g for 10 min to remove cell debris. Rinsed explants and ten 0.5 mL aliquots of pooled supernatant were then stored at −80°C. The remaining supernatant was centrifuged at 150,000×g for 1 hour at 4°C in a Beckman L8-80M ultracentrifuge. The resultant pellets were pooled and washed in PBS before finally being resuspended in 0.5 mL of PBS. Typical eSTBM preparations yield 0.2–0.4 mg vesicles. Five eSTBM preparations were pooled for use in all experiments.

### Preparation of red blood cell microvesicles

Peripheral blood taken from non-pregnant women was centrifuged to pellet red blood cells. Cells were resuspended in PBS/2 mM CaCl_2_ and treated with calcium ionophore (Sigma Aldrich, UK) for 1.5 hours at 37°C. Cellular debris was then removed by centrifugation at 2000×g for 10 minutes. Supernatants were centrifuged at 150,000×g and microvesicles washed in PBS and protein concentrations determined by a Pierce BCA assay and resuspended at 5 mg/ml in PBS prior to freezing at −80°C for use in stimulation assays.

### Blood Collection

20–30 ml blood samples, from pregnant and non-pregnant donors, were collected into sodium heparin anti-coagulant (10 U/ml) and PBMC were prepared by density gradient centrifugation over lymphoprep (Axis Shield Diagnostics, Cambridgeshire, UK). Cells were washed twice with PBS and consecutive centrifugation of 800×g and 200×g for 10 minutes.

### PBMC stimulation Assays

10^6^/ml PBMC in human serum media (RPMI 1640 supplemented with 10% human serum (Sera Laboratories International, West Sussex, UK), 1% penicillin-streptomycin (50 IU/ml and 50 µg/ml), 1% glutamine, MEM NEAA, sodium pyruvate and 50 nM 2-mercaptoethanol (Gibco Invitrogen, Paisley, UK)) were incubated with varying concentrations of STBM preparations for 20 hours at 37°C/5% CO_2_. Samples were centrifuged at 10,000×g for 30 seconds to remove cellular debris and supernatants frozen at −80°C until assayed.

### Cytokine Arrays

Cytokine arrays were performed using the Human Cytokine Array Panel A Proteome Profiler (R&D Systems, Minneapolis, USA). Briefly, 0.5 ml supernatant from stimulated PBMC was incubated with the array and cytokines detected following manufacturer's instructions. Dots were detected using X-ray film and films scanned for pixel density analysis using ImageJ software.

### Cytokine ELISA

IL-1α, IP-10, TNFα, MIP-1α, IL-8, G-CSF and IL-6 ELISA kits were from Peprotech (NJ, USA) and IL-1β from BD Biosciences (Oxford, UK) and used following the manufacturer's instructions. 100 µl supernatant was analysed in duplicate, and for the IL-8, IL-6 and IL-1β ELISAs some samples were diluted in human serum media between 5- and 100-fold in order for the analyte concentration to fall within the standard curve range. Standards were prepared in human serum media, and matched non pregnant and normal pregnant samples were run on the same 96-well MaxiSorp Plate (Nunc, Denmark). In addition, 50 µg/ml concentrations of mSTBM and pSTBM were analysed, neither of the preparations contained detectable levels of cytokines. ELISA were developed using 2-2′-Azino-bis(3-ethylbenzthiazoline-6-sulphonic acid) Liquid Substrate System for ELISA (Sigma Aldrich, UK) and absorbance at 405 nm detected using a FLUOstar Optima (BMG Labtech) plate reader.

### Intracellular Cytokine Staining for Flow Cytometry

10^6^ PBMC from non-pregnant donors (n = 3) were stimulated for 20 hours with 50 µg/ml pSTBM in the presence of 3 µg/ml Brefeldin A (eBioscience, San Diego, U.S.A.), with 1 µg/ml LPS (Sigma Aldrich, UK) or left untreated. Cells were harvested and washed ×3 in PBS/2% FCS then fixed using Fixation buffer (eBioscience) prior to staining for 20 minutes at 4°C with CD14-Alexa647 (BioLegend, San Diego, U.S.A.) or CD19-PeCy7 (BD Biosciences, Oxford, UK). Cells were then washed and treated with Permeabilization buffer (eBioscience) following manufacturers instructions prior to incubation for 20 minutes at 4°C with FITC conjugated antibodies towards IL-6, IL-8 and IL-1β or PE conjugated antibody towards TNFα (BD Bioscience) or appropriate isotype control. Cells were washed and analysed immediately by flow cytometry on a LSR-II flow cytometer (BD Biosciences, Oxford, UK) and data analysed using FACS DIVA software (BD Biosciences, Oxford, UK).

### STBM Binding Assay

10^6^/ml PBMC in human serum media were incubated with 50 µg/ml pSTBM for 1 hour at 37°C, cells were then washed ×2 with PBS/2% FCS by centrifugation at 200×g for 5 minutes. Cells were stained with the following fluorescently labelled antibodies CD14-APC (eBiosciences), CD3-PeCy5 (Biolegend), CD19-FITC (Serotech), CD56-Pe-Cy7 and CD16-APC-Cy7 (BD Pharmingen) and NDOG-2-PE or IgG-PE isotype control. NDOG-2 is a trophoblast specific antibody that recognises placental alkaline phosphatase [19]. NDOG-2 or IgG control antibodies were conjugated to phycoerythrin (PE) using a lightning-link kit (Innova Biosciences, Cambridge, UK). Data was acquired immediately.

### STBM Internalisation study by Image Stream – imaging flow cytometry

5 mM stock solution of BODIPY FL Maleimide (Invitrogen, Paisley, UK) was filtered through a 0.5 ml 10 K Amicon Ultra Centrifugal Filter (Millipore, MA, U.S.A.) and used to label pSTBM. 0.5 ml pSTBM (5 mg/ml) were incubated with BODIPY FL Maleimide for 15 minutes at room temperature, 98% labelling of pSTBM was confirmed by flow cytometry. pSTBM were washed with 11 ml PBS with ultracentrifugation at 100,000×g, to remove free BODIPY FL Maleimide. As a control the same amount of BODIPY FL Maleimide and 100 µl 300 nm polystyrene beads (Duke Scientific, Palo Alto, U.S.A.) was subjected to the same ultracentrifugation wash. Post ultracentrifugation supernatant was removed and beads or pSTBM were suspended in 0.5 ml PBS, and a BCA assay performed to determine the pSTBM yield. To test all free BODIPY FL Maleimide was removed, 50 µg pSTBM or corresponding volume of bead control was incubated with 10^6^/ml PBMC. No labelling of the cells was detected with the control (data not shown).

10^6^ PBMC/ml were incubated with 50 µg/ml pSTBM-BODIPY FL Maleimide for t = 1, 6 or 20 hours then washed and fixed in PBS/1% paraformaldehyde. Monocytes were identified by size, and B cells were stained with directly conjugated PE-fluorescent antibody towards CD19 before analysis by Image Stream (Amnis, Seattle, U.S.A.). Data was analysed with IDEAS 4.0 software, using the internalisation application. Briefly, single cell, in focus images and BODIPY FL positive images alone were analysed (i.e.: only cells that have bound pSTBM were analysed). External versus internal BODIPY FL was plotted on a histogram with values less than zero deemed external pSTBM and greater than 1 considered to be internalised pSTBM.

### HLA-DR Staining

10^6^ PBMC/ml were stimulated with 50 µg/ml pSTBM for 20 hours, or left untreated, and then cells stained with directly conjugated fluorescent antibodies HLA-DR-PeCy7 (BD Bioscience), CD19-PE and CD14-Alexa647 (BioLegend). Mean fluorescence intensity of HLA-DR was determined by flow cytometry.

### Statistical Analysis

Differences in cytokine production between non-pregnant and normal pregnant women were determined by a Wilcoxon signed rank test. Significant differences between cytokines produced in response to varying levels of STBM within the same non-pregnant or normal pregnant group were sought using a non-parametric ANOVA Kruskal-Wallis test with Dunns post test. Differences in the amount of internalisation over time were found by a repeated measures ANOVA with Dunns post hoc test. Analyses were performed with Prism software.

## Results

**Figure 1:**
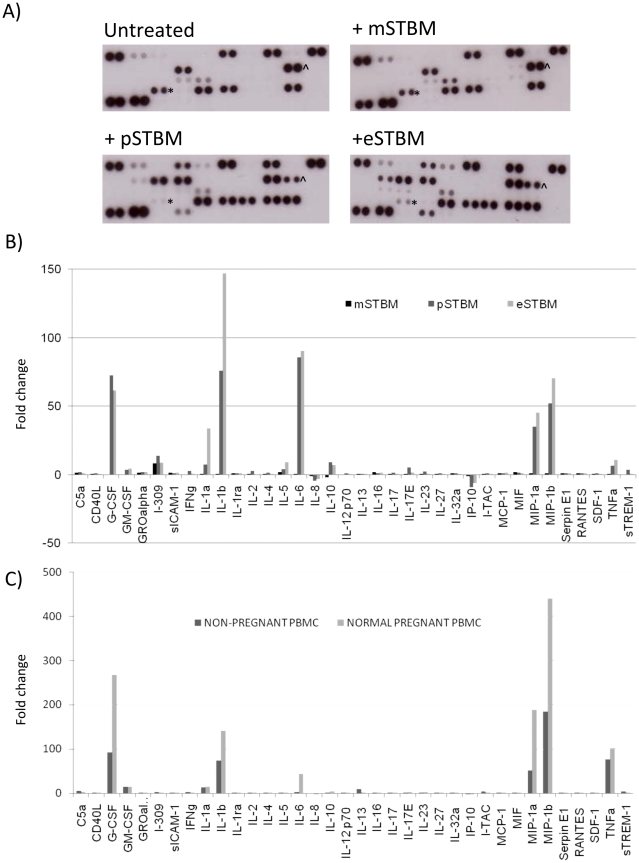
Regulation of cytokine production by mSTBM, pSTBM and eSTBM. PBMC from a non pregnant or normal pregnant donor were treated with STBM and cytokine production analysed using arrays. Briefly, 10^6^ PBMC/ml were treated with 50 µg/ml mSTBM, pSTBM or eSTBM for 20 hours at 37°C. Supernatants were harvested and cytokine production assessed by cytokine array analysis (R&D systems). A) Representative dot blots are shown for untreated cells and with STBM treatments, IP-10 (*) and IL-8 (∧) are highlighted. B) Graphical display of cytokines produced by PBMC from a non pregnant donor with treatment with mSTBM, pSTBM or eSTBM. C) Treatment of PBMC from a non pregnant or normal pregnant donor with pSTBM. Graphs display cytokines in the order on the dot blots, from top left to bottom right, excluding the positive control three standard pairs of dots on the top left, bottom left and top right.

PBMC from a non pregnant woman were incubated with 50 µg/ml mSTBM, pSTBM or eSTBM for 20 hours at 37°C, and supernatants used for cytokine array analysis, figure 1A. 36 cytokines were analysed and although the array is not quantitative the plots obtained indicate the trends of cytokine production. Pixel density intensity was determined and used to identify the fold change in cytokine concentration, comparing untreated PBMCs to treatment with each STBM preparation, figure 1B. Several cytokines were upregulated when incubated with pSTBM or eSTBM, but not mSTBM. Of note, MIP-1α, MIP-1β, IL-1α, IL-1β, IL-6 and G-CSF were increased by more than 20-fold. Smaller changes were also seen for TNFα, IL-10, I-309 and IL-5. Also, two cytokines were inhibited by incubation with pSTBM or eSTBM – IP-10 and IL-8. The array was repeated to determine the effect of pSTBM on an additional non-pregnant and a normal, third trimester pregnant blood donor, figure 1C. Consistent induction of TNFα, MIP-1α, MIP-1β, IL-1α, IL-1β, IL-6 and G-CSF was detected. The PBMC from the pregnant donor consistently produced greater levels of most cytokines than from the non-pregnant donor. IP-10 and IL-8 cytokine release was also inhibited several fold in the presence of pSTBM. As pSTBM and eSTBM induced a similar profile of cytokine production, we chose to use pSTBM in further experiments as only this method of preparation gives the high yield of microvesicles required for the study. mSTBM were included as a negative control.

**Figure 2:**
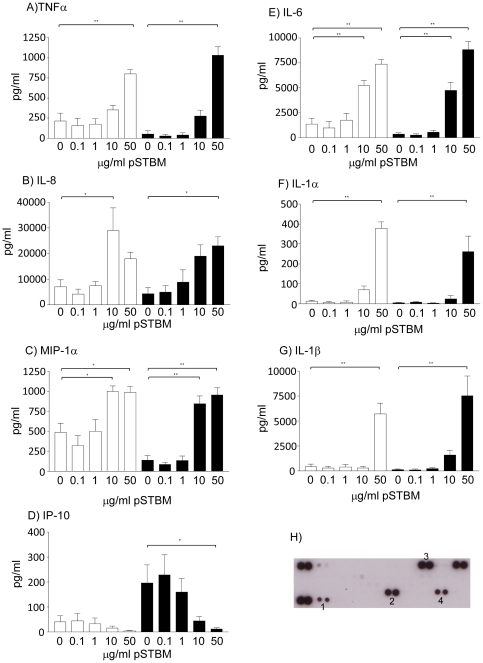
pSTBM alter cytokine production by PBMC from non and normal pregnant women. 10^6^/ml PBMC from 10 non pregnant (light bars) or 10 pregnant women (black bars) were treated with varying concentrations of pSTBM for 20 hours at 37°C. Supernatants were harvested and cytokine production assessed by ELISA for A) TNFα, B) IL-8, C) MIP-1α, D) IP-10, E) IL-6, F) IL-1α, and G) IL-1β (pg/ml). H) Cytokines and chemokines expressed by the pSTBM alone were determined by cytokine array, cytokines present above background levels in the human serum media were 1-RANTES, 2-MIF, 3-sICAM-1 and 4-Serpin E1.

Analysis of pSTBM alone showed the presence of RANTES, MIF, Serpin E1 and sICAM-1 on the vesicles, figure 2H. ELISAs were also performed on the pSTBM alone for the seven cytokines chosen for further study. None contained detectable levels of these cytokines (data not shown). Incubation of PBMC with 50 µg/ml microvesicles derived from red blood cells did not induce any changes to cytokines expression profiles (data not shown).

**Figure 3:**
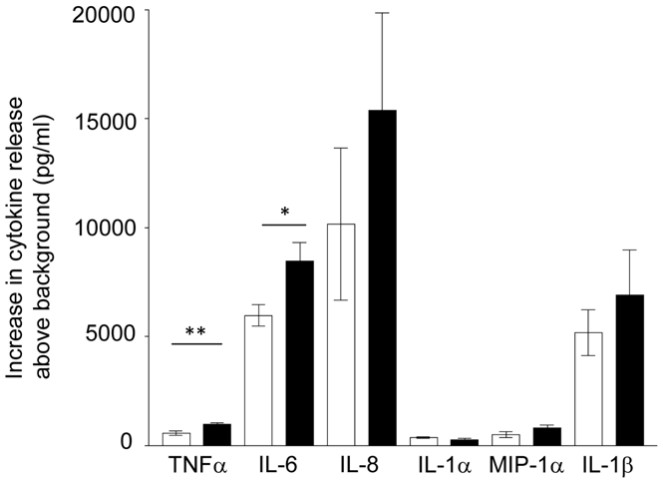
PBMC from normal pregnant women produce more TNFα and IL-6 in response to pSTBM than non pregnant women's PBMC. 10^6^/ml PBMC from non pregnant (light bars) or pregnant women (black bars) (n = 10) were treated with 50 µg/ml pSTBM for 20 hours at 37°C. Supernatants were harvested and cytokine production assessed by ELISA for TNFα, IL-6, IL-8, IL-1α, MIP-1α and IL-1β (pg/ml). Values calculated as the mean of value from cells treated with 50 ug/ml pSTBM minus background cytokine production.

From analysis of the array results TNFα, MIP-1α, IL-1α, IL-1β, IL-6, IP-10 and IL-8 were chosen for further investigation as these cytokines were consistently altered in the three cytokine array experiments. The number of cytokines that could be studied was limited by constraints on volumes of blood that could be taken, therefore IL-10, I-309 and IL-5 which showed the smallest changes were not be included. Technical difficulties with an ELISA for G-CSF meant that this cytokine could not be analysed further, and we studied only one of the MIP proteins as they have similar roles, MIP-1α was chosen as it has a more potent chemoattractant activity [20]. Ten matched non pregnant and normal pregnant women in their third trimester of pregnancy (range 35+3 to 39+6) were recruited and PBMC isolated for stimulation with mSTBM (50 µg/ml) or pSTBM at varying concentrations ranging from 50 to 0.1 µg/ml. Cells were incubated with STBMs, or LPS as a positive control (data not shown), for 20 hours at 37°C and supernatants harvested for ELISAs. TNFα, MIP-1α, IL-1α, IL-1β and IL-6 were all significantly induced by 50 µg/ml pSTBM in both non pregnant and normal pregnant groups, figure 2A, C, E–G. In addition, IL-6 and MIP-1α were significantly induced at 10 µg/ml; lower concentrations were not significantly stimulatory, figure 2C and 2E. pSTBM induced IL-8 cytokine production when detected by ELISA, in contrast to the cytokine array profile. This may be due to the high dose “hook effect” where a high target protein level combined with insufficient quantities of antibodies gives a false negative result. IL-8 was significantly induced at 10 µg/ml, but not 50 µg/ml, in the non pregnant group and 50 µg/ml in the normal pregnant group, figure 2B. IP-10 production was however inhibited by the presence of pSTBM, figure 2D. We found that the basal production of IP-10 was greater in the normal pregnant group, perhaps highlighting the heightened inflammatory nature of pregnancy, and a dose of 50 µg/ml pSTBM was able to significantly inhibit IP-10. A trend of inhibition is noted for the non pregnant group, however as the basal levels are low this did not achieve statistical significance. mSTBM were also incubated with PBMC but even at a dose of 50 µg/ml there was no significant effect on any of the cytokines studied, in both non pregnant or normal pregnant women, table 1. pSTBM (50 µg/ml) stimulation, above basal production, of TNF-α, IL-6, IL-8, IL-1α, MIP-1α and IL-1β in both the non pregnant and normal pregnant groups is shown in figure 3. For all cytokines, except IL-1α, a trend of greater cytokine induction is noted within the normal pregnant group, while induced levels of both TNFα and IL-6 were significantly higher from normal pregnant women's PBMC compared to non-pregnant.

**Table 1:** Cytokine production is regulated by pSTBM but not mSTBM.

		Cytokine production						
Donors	Treatment	TNFα	IL-6	IL-8	IL-1α	MIP-1α	IP-10	IL-1β
Non-pregnant	pSTBM	979.1 (88.5)	8487 (835.7)	15390 (4467)	255.8 (79.1)	812.8 (135.1)	−184 (68.2)	6919 (2070)
Non-pregnant	mSTBM	−7.373 (8.8)	−132.7 (89.5)	1040 (762)	−2.026 (1.1)	−9.609 (62.5)	5.25 (21.6)	−102.8 (82.8)
Pregnant	pSTBM	583.9 (110.9)	5980 (495.5)	10160 (3494)	365.6 (29.1)	497.5 (129.8)	−36.24 (22.8)	5184 (1053)
Pregnant	mSTBM	2.184 (49.6)	−140.8 (363)	−339.9 (1182)	−2.981 (4.0)	−211.3 (119.2)	−16.53 (12.0)	−44.5 (74.8)

10^6^/ml PBMC from non pregnant or pregnant women were treated with 50 µg/ml pSTBM or mSTBM for 20 hours at 37°C. Supernatants were harvested and cytokine production assessed by ELISA for TNFα, IL-6, IL-8, IL-1α, MIP-1α, IP-10 and IL-1β (pg/ml). Values shown are change from mean background production, from 10 non pregnant or 10 pregnant women, standard error in brackets.

**Figure 4:**
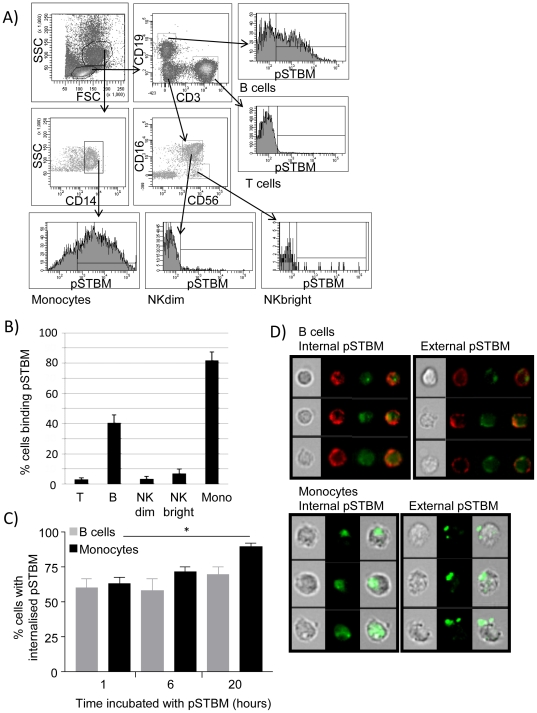
pSTBM bind to monocytes and B cells. 10^6^ PBMC from normal pregnant women were incubated with 50 µg/ml pSTBM for one hour at 37°C. Cells were then washed in PBS and stained for antibodies towards CD3, CD14, CD19, CD56 and CD19, and the proportion of cells with bound pSTBM identified with the trophoblast specific marker NDOG-2. Gates were set by staining PBMC with all six antibodies without the addition of pSTBM. NDOG-2 staining of cell populations was also compared to an isotype control (representative staining profile shown in A). Binding of pSTBM to cell populations shown in B (n = 3; mean +/− S.E.M.). C) Image stream analysis of B cell (grey bars) and monocyte (black bars) internalisation of pSTBM (labelled with BODIPY FL Maleimide) with IDEAS software (n = 3; mean +/− S.E.M.; * = p<0.05). D) Representative images of cells with internal or external pSTBM - B cells showing brightfield image, CD19 (red), pSTBM (green) and overlay (top panel) and monocytes with brightfield image, pSTBM (green) and overlay (bottom panel).

Next, we determined the binding profile of pSTBM to PBMC by using six colour flow cytometry. We examined the T cell, B cell, NKdim, NKbright and monocyte cell populations of normal pregnant women after incubation with pSTBM for 1 hour. STBM were identified using the trophoblast specific antibody NDOG2. PBMC were stained with antibodies towards CD14, CD3, CD19, CD56 and CD16 to identify T cells, B cells, monocytes, NKdim (CD56+CD16+) and NK bright (CD56brightCD16-) cells, figures 4A. pSTBM binding was detected on small numbers of T and NK cells, and to a greater extent to B cells (40%) and Monocytes (82%), figure 4B. Next we used Image Stream (imaging flow cytometry) and IDEAS analysis software to determine if pSTBM were phagocytosed by the monocytes and B cells. pSTBM were labelled with BODIPY FL maleimide dye and pSTBM incubated with 3×10^6^ PBMC from non-pregnant donors (n = 3). Cells were incubated with pSTBM for 1, 6 or 20 hours then images acquired by Image Stream. Briefly, image stream captures fluorescence microscopy images of cells as the suspension of cells passes through the machine, which enables statistical analysis of pSTBM internalisation on a large number of cells, something which is not possible by conventional confocal microscopy. We found that after one hour of incubation with pSTBM, approximately 60% of B cells and monocytes that had bound pSTBM had phagocytosed the vesicles. Over time, monocytes continued to internalise pSTBM, 90% of monocytes had internalised the pSTBM after 20 hours incubation, whereas no more B cells internalised vesicles, figure 4C. Representative images of B cells and monocytes with internalised or external pSTBM are shown in figure 4D.

**Figure 5:**
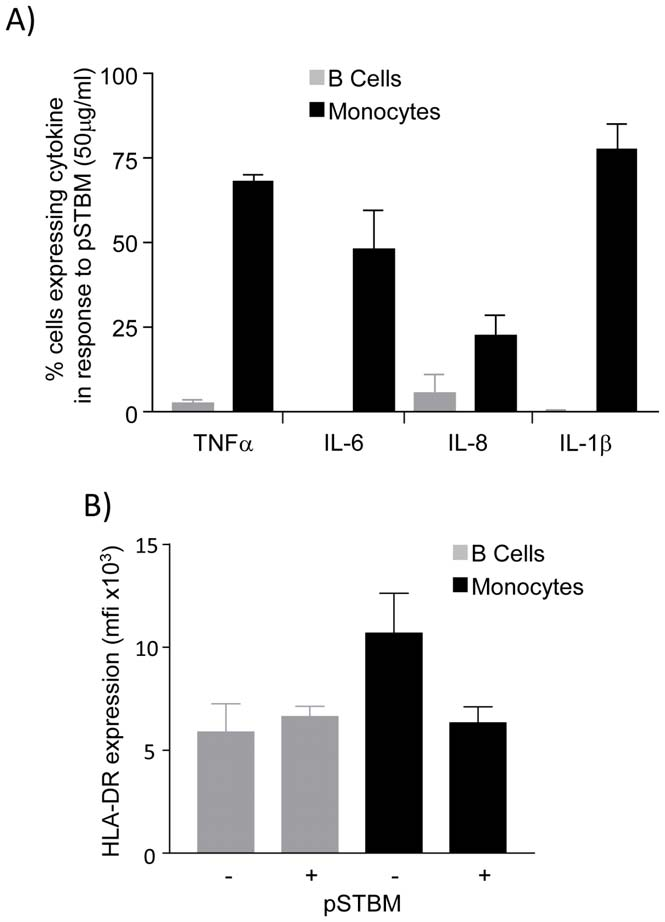
Monocytes, not B cells, produce the cytokines IL-6, IL-8, TNFα and IL-1β and downregulate HLA-DR in response to pSTBM. A) 10^6^/ml PBMC from non-pregnant donors (n = 3) were stimulated with 50 µg/ml pSTBM, or left untreated, for 20 hours in the presence of Brefeldin A. Intracellular cytokine analysis was performed to detect production of TNFα, IL-6, IL-8 and IL-1β from either B cells (grey bars) or monocytes (black bars). Data shown is the increase in proportion of cells expressing each cytokine above cytokine production in untreated samples, mean (+/− S.D.). B) pSTBM binding to monocytes, but not B cells, caused down-regulation of HLA-DR, shown by median fluorescence intensity of HLA-DR antibody staining (C), (n = 3; mean +/− S.D.).

Finally, to identify which of the PBMC were producing the most abundant cytokines, we performed intracellular cytokine staining on pSTBM stimulated PBMC from non-pregnant donors. The monocytes were the major producers of IL-6, IL-8, IL-1β and TNFα, whereas only a few B cells (2–6%) were primed to produce IL-8 and TNFα, figure 5A. HLA-DR expression was also analysed as up-regulation indicates activation of antigen presentation capacity. Surprisingly, HLA-DR expression was downmodulated on the surface of monocytes, but not B cells, after 20 hours incubation with pSTBM, figure 5B.

## Discussion

Previously, we have shown that pSTBM, but not mSTBM, induced PBMC from non-pregnant donors to produce inflammatory cytokines (TNF-α, IL-18, and minimal amounts of IL-12p70 and IFN-γ) [10]. Here, we have used cytokine arrays to extend this study and have identified that a wider range of pro-inflammatory cytokines are induced by pSTBM as well as one (IP-10) which is inhibited. Furthermore, for some of the cytokines, there were significant differences in their production in pregnant compared to non-pregnant women. IL-18 is not included on this array, therefore previous observations of IL-18 induction were not confirmed. Also, neither IL-12p70 nor IFN-γ were altered on the cytokine arrays, which may be due to the sensitivity of the array, however on the basis of this result we decided not to include them in the further experiments in favour of those cytokines more greatly altered.

Interestingly, we showed that for the 36 cytokines studied the profile was very similar for eSTBM and pSTBM, but mSTBM were not as stimulatory. This is consistent with a recent report that pSTBM and eSTBM but not mSTBM induce monocyte production of IL-6, IL-8 and IL-1β [21]. We found that pSTBM significantly induce PBMC production of TNFα, MIP-1α, IL-1α, IL-1β, IL-6, and IL-8, whether the donors were pregnant or not. However, mSTBM were not stimulatory. To generate mSTBM trophoblast tissue is mechanically removed from villi, tissue is suspended in salt buffer at 4°C and released microvesicles are collected from the supernatant. We believe this is less likely to produce biologically normal vesicles than placental perfusion or explant cultures, which are produced under more physiological conditions (O_2_, temperature, buffered solutions). We are currently performing proteomic analysis to compare mSTBM and pSTBM and this will be reported in a subsequent paper.

Detecting circulating levels of cytokines is notoriously difficult and often conflicting reports have been published citing the increased or decreased quantities, or absence, in pregnancy. The six cytokines investigated here are shown in some studies to be elevated in the peripheral blood of pregnant women, compared to non pregnant women, or are found at increased concentrations locally at the placenta and are therefore considered important for successful pregnancy. Total IL-1 concentrations have been reported to increase in the serum of pregnant women during the second and third trimester and are significantly higher than in non pregnant women, but whether this was IL-1α or IL-1β was not defined [22]. Together with TNFα, the IL-1 proteins IL-1α and IL-β are pro-inflammatory, and TNFα can also be detected at increased levels in pregnant women [23]. MIP-1α is a chemokine, also known as CCL3, and is produced by first trimester placenta cytotrophoblast to aid recruitment of monocytes and natural killer cells to the decidua to enable efficient placentation [24]. MIP-1α instigates acute and chronic inflammatory host responses by recruiting pro-inflammatory cells expressing its receptor CCR5 to the sites of injury or infection [25]. Finally, IL-8, which is also a chemokine, functions as a chemoattractant and angiogenic factor, and hence helps drive immune responses. Taken together the induction of all these cytokines (TNFα, IL-1α, IL-β and MIP-1α) in a pregnant woman would lead to a generalised inflammatory status. To counteract this we have noted that anti-inflammatory cytokines are also modulated by STBM, which may prevent excessive inflammation. IL-6 levels are raised in the peripheral blood of pregnant women [22], IL-6 has both pro-inflammatory and anti-inflammatory functions and therefore its presence in pregnancy may be to prevent excessive immune activation as it inhibits both IL-1 and TNFα. Similarly, inhibition of IP-10 would be immunomodulatory, preventing type 1 cytokine release.

Placentas exert various immunomodulatory effects [26], and the shedding of STBM which could carry regulatory placental proteins, lipids or nucleic materials extends the immunomodulatory capacity into the maternal system. Immunomodulation of T cell responses have been previously described [15]; STBM inhibition of allogeneic immune responses (MLR) is attributed to syncytiotrophoblast membrane glycoproteins [14] and inhibition of PHA/ionomycin activation of T cells is dependent upon microvesicle expression of FasL (where T cell Fas expression is high, for example on the Jurkat cell line) and PD-L1, the ‘programmed cell death 1 ligand 1’ immunosuppressive molecule [27], [28]. In our study we observe that pSTBM are able to inhibit IP-10 production. IP-10 is a member of the CXC chemokine family, also known as CXCL10, and is involved with monocyte, T cell, natural killer (NK) cell and dendritic cell chemoattraction, as well as promotion of T cell adhesion to endothelium [29]. It is induced by pro-inflammatory stimuli and is associated with the pathogenesis of various diseases, such as diabetes mellitus type 1 [30] as well as allograft rejection [31]. It is also an anti-angiogenic factor [32] and is associated with the development and continuation of Th1 responses; as its name suggests, the interferon-inducible protein 10 KDa (IP-10) is induced by the classical type 1 cytokine IFNγ, and its receptor, CXCR3, has higher expression on Th1 than Th2 cells [33], [34]. Concentrations of serum IP-10 have been found to be significantly higher in normal pregnant women than non pregnant women [35], and even higher levels circulate in pre-eclamptic women. In line with this, we detect more IP-10 production by PBMC from pregnant women than non-pregnant women. Gotsch *et al* (2007) propose that elevated maternal serum IP-10 contributes to the anti-angiogenic state of pre-eclampsia (along with sFlt-1 and endoglin). Here we suggest that in normal pregnancy STBM are able to reduce the levels of IP-10 produced by PBMC, thereby enabling angiogenesis and skewing of immunity to type 2 responses that are important for healthy pregnancy. Finally, the cytokine arrays indicate STBM induction of G-CSF. This factor aids pregnancy success in patients with recurrent miscarriage or repetitive failed implantation in IVF cycles [36], [37] and has been shown to be induced by trophoblast derived microvesicles in vitro [38], further indicating that STBMs may be important for healthy pregnancy.

We have shown that pSTBM bind monocytes, and to a lesser extent B cells. In addition, we have shown that monocytes and B cells are able to rapidly phagocytose pSTBM. This suggests that the receptor for pSTBM on monocytes and B cells may be involved with phagocytosis as well as binding. Possible receptors include Toll-like Receptors, Receptor for Advanced Glycation End Products (RAGE) and Integrins. Further work is in progress to assess the nature of this interaction.

While pSTBM bind to both monocytes and B cells, it appears that the monocytes are responsible for the production of the majority of the most abundantly produced cytokines (TNFα, IL-6, IL-8 and IL-1β). Even though monocytes are stimulated to produce cytokines they do not up-regulate HLA-DR expression, and HLA-DR expression is reduced by monocytes stimulated by pSTBM. Increased HLA-DR expression upon antigen stimulation aids antigen presentation and hence T cell stimulation. T cells are not normally activated in pregnancy, and no placental derived antigenic peptide has been reported. However down-modulation of class II molecules would reduce the potential for antigen presentation and prevent T helper cell stimulation.

The minimal binding capacity of pSTBM to T cell and NK cells, may suggest that only a small proportion of these cells may be subjected to direct immunomodulation by placental microvesicles. As mentioned above, allogenic or mitogen induced T cell responses are inhibited by STBM, and NK cells are also reported to be subjected to STBM derived immunosuppression through MIC/ULBP protein binding to their NK receptor NKG2D [39]. It is unclear why our preparations are not binding to NK cells at greater levels.

PBMC from normal pregnant women showed a significantly increased production of TNFα and IL-6 when compared to the responses from non-pregnant women. IL-8, IL-1β and MIP-1α also showed increased production but not to levels of significance. This suggests immune cells are primed to respond to STBM by pregnancy which supports our previous observations of enhanced monocyte activity in pregnant compared to non-pregnant women [40]. The mechanism of this is unknown, but it could be due to the increased levels of pro-inflammatory cytokines and hormones released by the placenta, or prior exposure to STBM in vivo.

In conclusion, we have shown immune cells produce of a variety of proinflammatory cytokines in response to STBM stimulation, which may contribute to the increased inflammation seen in normal pregnancy. Interestingly we have also found that constitutive IP-10 production is inhibited by STBMs, which would encourage skewing of the immune system away from excessive type 1 cytokine responses in normal pregnancy.
